# High-resolution single-cell analysis paves the cellular path for brain regeneration in salamanders

**DOI:** 10.1186/s13619-022-00144-5

**Published:** 2022-10-19

**Authors:** Binxu Yin, Xinyun Li, Gufa Lin, Heng Wang

**Affiliations:** 1 College of Animal Science and Technology, Huazhong Agricultural University, Wuhan, China; 2 Key Laboratory of Spine and Spinal Cord Injury Repair and Regeneration of Ministry of Education, Orthopaedic Department of Tongji Hospital, School of Life Sciences and Technology, Tongji University, Shanghai, China

## Abstract

Salamanders are excellent models for studying vertebrate brain regeneration, with the promise of developing novel therapies for human brain lesions. Yet the molecular and cellular mechanism of salamander brain regeneration remains largely elusive. The insight into the evolution of complex brain structures that lead to advanced functions in the mammalian brain is also inadequate. With high-resolution single-cell RNA sequencing and spatial transcriptomics, three recent studies have reported the differentiation paths of cells in the salamander telencephalon in the journal *Science*, bringing both old and new cell types into the focus and shedding light on vertebrate brain evolution, development, and regeneration.

## Main text

The human brain’s ability to regenerate is almost nil. When our brain suffers damage caused by traumatic injury, stroke, Parkinson’s and Alzheimer’s diseases, etc., it generally results in impairments in consciousness, sensory, motor, and memory functions. Treatment options are limited, costly, and insignificant to overcome functional defects. The urodele amphibian salamanders, however, have regenerative capacities that are the envy of mammals, including regeneration of the brain (Tanaka and Ferretti 2009). Similar to the fish (Lange and Brand 2020), injury in the salamander telencephalon elicits a neurogenic response and activation of radial glia-like progenitor cells (or ependymoglial cells, EGCs), to replenish the cells lost in the lesion area. EGCs are the predominant glial cells in the central nervous system of salamanders and give rise to many cell types in the brain during development (Joven and Simon 2018). However, it remains unclear how EGCs are activated, and whether and how the full spectrum of cell types is restored during brain regeneration. Three recent papers published in *Science* have addressed the cellular dynamics of brain development and regeneration in the newt (*Pleurodeles waltl*) (Woych et al. 2022) and the axolotl (*Ambystoma mexicanum*) (Lust et al. 2022; Wei et al. 2022).

All three papers started with single-cell level analyses of the cell types in salamander brain development, and addressed the similarities between brain regeneration and development. Single-cell RNA sequencing (scRNAseq) analysis allows the identification of new source of cells, or new cell types generated from previously known progenitor cells. Woych et al. and Lust et al. demonstrated the existence of cross-species conservation of brain cells in salamanders. The neuronal diversity in the salamander brain is not decreased, despite the reduced complexity of general brain anatomy compared to higher vertebrates. For instance, Lust et al. found 29 clusters of glutamatergic neurons and 30 clusters of γ-aminobutyric acid–releasing (GABAergic) neurons in the axolotl telencephalon. Woych et al. found 47 clusters of glutamatergic neurons and 67 clusters of GABAergic neurons in the newt telencephalon. Both studies showed a much greater degree of complexity and diversity of neuronal cell types in the salamander than expected.

The cell-type atlas built from the scRNAseq analysis also facilitates the comparison of cell types across tetrapod species. The transition from aquatic to terrestrial vertebrates presents new challenges and opportunities for the transformation of many different parts and organs in the body (MacIver and Finlay 2022). Among them, the brain gained an advantage over other organs, as evidenced by an increase in relative size and a more complex and refined partitioning (Heldstab et al. 2022). Woych et al. compared the telencephalon neuron types across different species and revealed that the sub-pallial regions in the amphibian may have evolved into different parts of the brain in higher vertebrates. Overall, the mammalian subiculum and entorhinal cortex (Yao et al. 2021), but not the neocortex, can be traced back to the salamander dorsal pallium neurons. In contrast, the salamander ventral pallium can only expand to the anterior dorsal ventricular ridge (aDVR) in reptiles. The existence of regions similar to the amphibian ventral pallium and reptile aDVR in mammals remains mysterious.

**Fig. 1:**
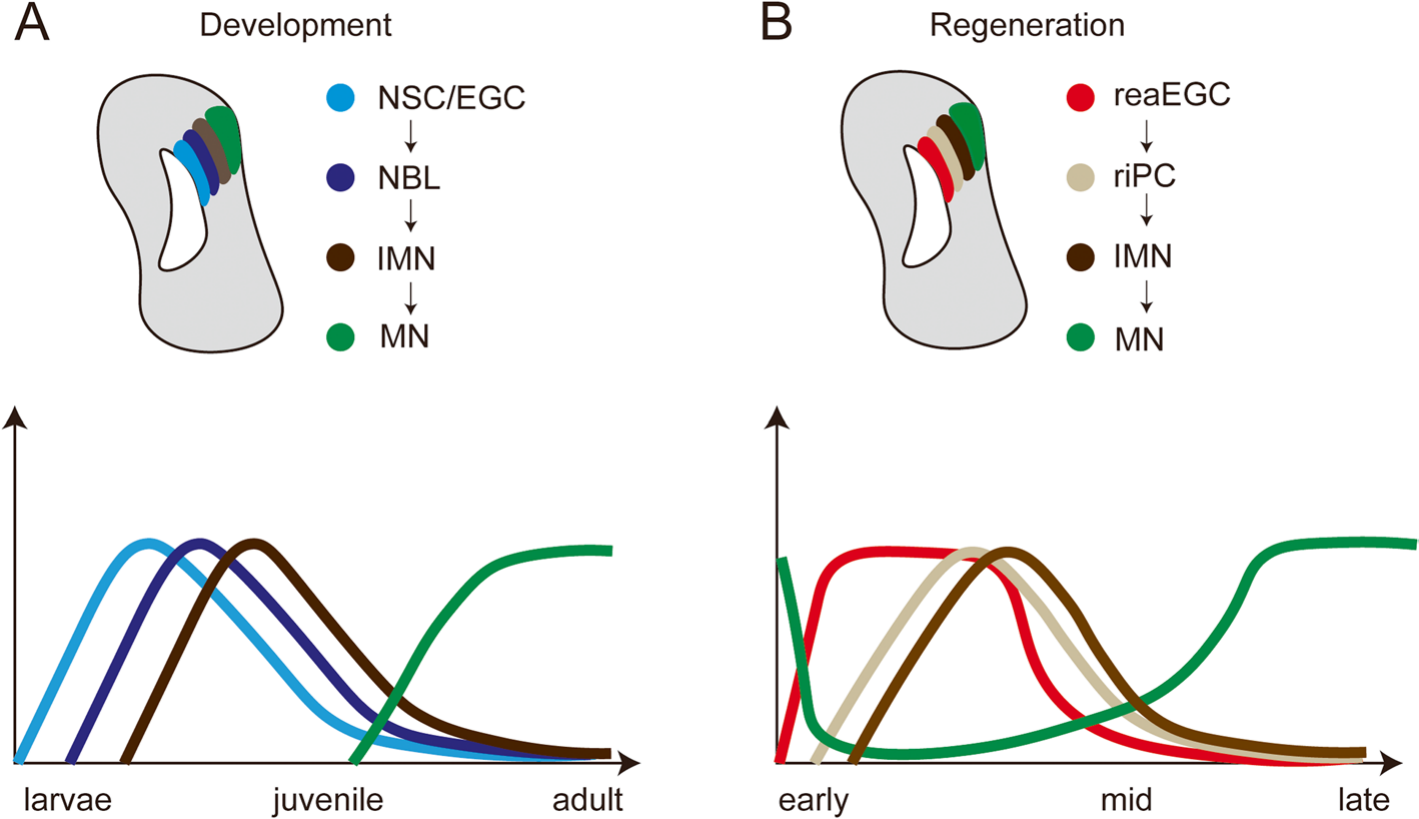
Dynamics of ependymoglial cells, neuronal progenitors and neurons during axolotl brain development and regeneration. NSC, neural stem cell; NBL, neuroblast; EGC, ependymoglial cell; reaEGC, reactive ependymoglial cell; riPC, regeneration intermediate progenitor cell; IMN, immature neuron; MN, mature neuron

The authors continued to explore the cellular dynamics during brain development and regeneration, and revealed that a type of EGCs, rather than neural stem cells, are the major contributor to brain regeneration in salamanders. Lust et al. found three main types of EGCs in the axolotl telencephalon: quiescent, active, and pro-neuro. These cells are widely and evenly distributed in all anatomical regions of the telencephalon. Wei et al., went a step further to use a new method called Stereo-seq to analyze the dynamics and diversities of EGCs at high spatial resolution. The technique combines DNA nanoball sequencing technology with in situ RNA capture, allowing for extensive identification of subcellular-level transcripts with spatial information. They found a major class of developmentally-related EGCs (dEGCs) present in the ventricular zone at stage 44 axolotl telencephalon, but begin to decrease after stage 54 and disappear at the juvenile stage. Lineage-tracing and trajectory analysis showed that these EGCs give rise to neuroblasts (NBLs), which further generate immature and mature neuron types (Fig. 1A).

Next, both Lust et al. and Wei et al. performed a mechanical injury in the pallium and their findings reiterated the prominent role of injury-induced EGCs in brain regeneration (Kirkham et al. 2014). Lust et al., found that early in brain injury, EGCs at the wound margin are the dominant cell population, with genes associated with wound healing and cell migration upregulated; neuroblasts are the most abundant cell type in the mid-regeneration stage; glutamatergic and GABAergic neurons are identified at regeneration hotspots in the later regeneration stages. The re-establishment of olfactory bulb input projections was found, indicating restoration of connections between the injured area and the distal brain regions. Importantly, regenerative neurogenesis recapitulates developmental neurogenesis, suggesting that existing regulatory programs can be reactivated to direct regeneration. Wei et al., also found that the neurogenesis programming during regeneration resembles the developmental process (Fig. 1B). They named the injury-induced EGCs as reactive EGCs (reaEGCs). reaEGCs express genes involved in cell proliferation, migration, and extracellular matrix remodeling. These cells then change into regeneration intermediate progenitor cells (riPCs). riPCs give rise to new immature neurons covering the edge of the injury, which subsequently generate intermediate and mature neurons and progress toward the center of the incision, a process accompanied by a transition in cell state from wound response to neuronal differentiation and a reduction in proliferative potential. The reaEGCs and dEGCs exhibit transcriptomic similarities. Since adult axolotls are already depleted of dEGCs at the time of injury, the reaEGCs may originate from local EGCs that express *Wnt8b* (wntEGCs) or *Sfrp1* (SfrpEGCs), or from other subtypes of resident EGCs located in the ventricular zone. Thus, these studies demonstrate the existence of injury-induced and so-called reactive EGCs for brain regeneration in the salamander. However, it remains unclear whether there is any consequence if these special EGCs are depleted during brain regeneration.

## Conclusions

Taken together, these studies have utilized state-of-the-art single-cell-omics techniques and cell labeling and tracing tools to capture the cellular diversity and dynamics during brain development and regeneration in salamanders. The findings lay the foundation for a deeper understanding of the evolution, structure, and function of vertebrate brains. As emerging model organisms, salamanders have unique attractions: their regenerative capacity is the strongest among vertebrates. Each of these studies combined the power of single-cell genomics and the uniqueness of regenerative salamanders, and produced massive data to expand our understanding of neuronal cell creation and regeneration. The newly identified pro-regenerative signals and cell types induced during salamander brain regeneration, such as the reactive EGCs in axolotl telencephalon, could have important implications for designing gene and cell therapies for neurodegenerative disorders in humans.

## Data Availability

Not applicable.
